# miR-152 Regulates Bovine Myoblast Proliferation by Targeting KLF6

**DOI:** 10.3390/ani11103001

**Published:** 2021-10-19

**Authors:** Chengchuang Song, Xue Fang, Zhaoxin Yang, Qi Wang, Fantong Meng, Yaqi Chen, Junhao Chen, Bei Zhao, Yanhong Wang, Xingtang Fang, Lihong Gu, Chunlei Zhang

**Affiliations:** 1 Institute of Cellular and Molecular Biology, School of Life Science, Jiangsu Normal University, Xuzhou 221116, China; chengchuangsong@jsnu.edu.cn (C.S.); fangxue@jsnu.edu.cn (X.F.); wangqiqi@jsnu.edu.cn (Q.W.); 1020180004@jsnu.edu.cn (F.M.); gigichen@jsnu.edu.cn (Y.C.); cjh1109@jsnu.edu.cn (J.C.); 3020192471@jsnu.edu.cn (B.Z.); yhwang@jsnu.edu.cn (Y.W.); 6019940077@jsnu.edu.cn (X.F.); 2 Key Laboratory of Animal Genetics, Breeding and Reproduction of Shaanxi Province, College of Animal Science and Technology, Northwest A&F University, Yangling 712100, China; yangzhx29@mail2.sysu.edu.cn; 3 Institute of Animal Science & Veterinary Medicine, Hainan Academy of Agricultural Sciences, Haikou 571100, China; nil2008@yeah.net

**Keywords:** miR-152, skeletal muscle, KLF6, proliferation

## Abstract

**Simple Summary:**

Our study found that miR-152 functions in bovine myoblasts to inhibit proliferation. Our prediction and experimental verification revealed that Kruppel-Like Factor 6 (KLF6) is a direct target gene of miR-152. Therefore, miR-152 and its target gene KLF6 have certain effects on the development of bovine skeletal muscles.

**Abstract:**

Though miRNAs have been reported to regulate bovine myoblast proliferation, but many miRNAs still need to be further explored. Specifically, miR-152 is a highly expressed miRNA in cattle skeletal muscle tissues, but its function in skeletal muscle development is unknown. Herein, we aimed to investigate the role of miR-152 in regulating bovine myoblast proliferation. Functionally, RT-qPCR, Western blotting, EdU assay, and flow cytometry detection results showed that miR-152 inhibited bovine myoblast proliferation. Mechanistically, we demonstrated transcription factor KLF6 was a target gene of miR-152 by means of bioinformatics software prediction and dual-luciferase report analysis, which had been demonstrated to be favorable for myoblast proliferation. Collectively, our research suggested that miR-152 inhibits bovine myoblast proliferation via targeting KLF6.

## 1. Introduction

Skeletal muscle development is a complex multistage process, which is directed initially from pluripotent stem cells to myogenic progenitor cells and then to mononuclear myoblasts [1,2]. Then, mononuclear myoblasts further proliferate and fuse to form multinucleated myotubes, which fuse to form muscle fibers, and finally develop into skeletal muscles with the contraction function. After birth, the number of muscle fibers generally does not increase anymore [3]. For livestock animals, the muscle cells count is the prerequisite to determine meat production. Therefore, it is of great significance to study the regulatory mechanism of myoblast proliferation. This process could be regulated by positive cell cycle regulators Proliferating cell nuclear antigen (PCNA) [4], cyclin-dependent kinases2 (CDK2) [5] and cyclin members. In addition, some non-coding RNAs like microRNAs (miRNAs) directly or indirectly regulate the expression of these genes, thereby affecting myoblast proliferation [6,7,8].

MiRNAs are a kind of small non-coding endogenous RNA with a length of about 22 nucleotides, which are evolutionally conserved, mainly binding to the 3′-UTR (untranslated region) region of the target genes, thereby inhibiting translation or degradation of the target gene at the posttranscriptional level [9]. Recently, some reports have confirmed that miRNAs play a vital regulatory role in myoblast proliferation. Muscle-specific miRNA miR-133 can promote myoblast proliferation by targeting the serum response factor (SRF) [10]; miR-128a inhibits myoblast proliferation through targeting the insulin receptor substrate 1 (IRS1)-dependent insulin signaling [11]; miR-499 promotes myoblast proliferation via targeting transforming growth factor β receptor 1 (TGFβR1) [12]. Besides these muscle-specific miRNAs, some non-muscle-specific miRNAs are also involved in the regulation of myoblast proliferation. For example, the miR-17-92 cluster (miR-17, -20a, and -92a) promotes myoblast proliferation by targeting actin-associated protein enigma homolog 1 (ENH1) [13]. miR-27a has been confirmed to promote myoblast proliferation via targeting myostatin [14]. Up to now, numerous studies on the regulation of miRNA for myoblast proliferation have been reported [15]. Nevertheless, there is still large space for research on the regulation of myoblast proliferation by miRNAs, especially in livestock animals.

In this study, we aimed to investigate additional potential miRNAs for bovine myoblast proliferation. We further analyzed the miRNA expression feature in skeletal muscles of cattle at different growth stages [16]. Among these miRNAs, miR-152 caught our attention, which is a differentially expressed miRNA in skeletal muscles of cattle at different growth stages, and its role in bovine skeletal muscle myogenesis remains undetermined. After a series of experiments, we demonstrated that miR-152 inhibits bovine myoblast proliferation by targeting KLF6, which is an important transcription factor for skeletal muscle development. The results will be helpful for further studies of skeletal muscle development in cattle.

## 2. Materials and Methods

### 2.1. Tissue Samples Collection

Ninety days of gestation fetal calf and 24-month-old cattle (Shaanxi Kingbull Livestock, Baoji, China) heart, liver, spleen, lung, kidney and skeletal muscle samples were collected. The tissue samples were used for RNA extraction and cell isolation. Animal care and study protocols were approved by the Animal Care Commission of School of Life Science, Jiangsu Normal University (permit number JSNUSK20200922-01).

### 2.2. Cell Culture and Transfection

The bovine myoblast cells were isolated from the longissimus or hind limb muscles of fetal bovine samples (Shaanxi Kingbull Livestock, Baoji, China). For detailed isolation methods, please refer to our previous research paper [17]. The isolated cell suspensions were inoculated into Petri dishes and cultured in a high-sugar medium containing 20% FBS and 1% penicillin/streptomycin at 37 °C in 5% CO_2_; miR-152 mimic and inhibitor were synthesized by RiboBio (Guangzhou, China). Lipofectamine 3000 transfection reagent (Thermo Fisher Scientific, Waltham, MA, USA) was used for mimic or inhibitor transfection according to the transfection reagent instructions.

### 2.3. RNA Extraction and Quantitative Real-Time PCR

The total RNA of tissues or cells was extracted with the Trizol reagent. Total RNAs were reverse-transcribed into cDNA by using a reverse transcription kit (AG, Accurate Biology, Changsha, China). For miR-152 expression analysis, the stem–loop RT primer containing partial complementary sequences of the miR-152 mature sequence was reverse-transcribed with a reverse transcriptase (AG, Accurate Biology). RT-qPCR was performed with a SYBR Green Kit (AG, Accurate Biology). The 2^−ΔΔCt^ method was used to calculate the relative expression level of mRNA or miRNA. The primer sequences are listed in Table S1.

### 2.4. Western Blotting

Bovine myoblast cell proteins were extracted using the RIPA buffer with 1% PMSF (KeyGEN BioTECH, Nanjing, China). The protein concentration was evaluated using a BCA kit (Beyotime, Shanghai, China). The proteins were separated with 12% SDS-PAGE and transferred to PVDF membranes. According to the manufacturer’s instructions, the membrane was incubated with the primary antibody and the secondary antibody successively. The membranes were exposed to the ECL kit(Solarbio, Beijing, China). The detailed process was performed as previously described. The primary antibody and the secondary antibody are listed in Table S2.

### 2.5. EdU Assay

Twenty hours after transfection of the miR-152 mimic or inhibitor, the circumstances of bovine myoblasts at the S stage were detected using a Cell-Light EdU Apollo 567 in vitro imaging kit (RiboBio, Guangzhou, China). The detailed operation process was performed according to the manufacturer’s instructions.

### 2.6. Cell Cycle Assay

Twenty-four hours after transfection of the miR-152 mimic or inhibitor, bovine myoblasts were processed using a cell cycle testing kit (Multisciences, Hangzhou, China), and flow cytometry was used to analyze the cell cycle. The detailed operation process was performed according to the manufacturer’s instructions.

### 2.7. Dual-Luciferase Reporter Assay

The KLF6 3′-UTR sequence containing the miR-152 binding site (wild-type or mutant-type) was inserted into the psi-CHECK-2 luciferase reporter plasmid according to our previous study. The wild-type or mutant type plasmid was co-transfected with the miR-152 mimic into HEK293T cell lines using the Lipofectamine 3000 transfection reagent. A Dual Luciferase Assay Kit (Promega, Madison, WI, USA) was used to detect firefly luciferase activity and *Renilla* luciferase activity successively according to the manufacturer’s instructions.

### 2.8. Statistical Analyses

The data are presented as the means ± SEM; *p*-values were calculated by means of a two-tailed Student’s *t*-test; *p* < 0.05 was considered statistically significant (* *p* < 0.05, ** *p* < 0.01).

## 3. Results

### 3.1. The Expression Characteristics of miR-152 in Cattle Tissues

**Figure 1:**
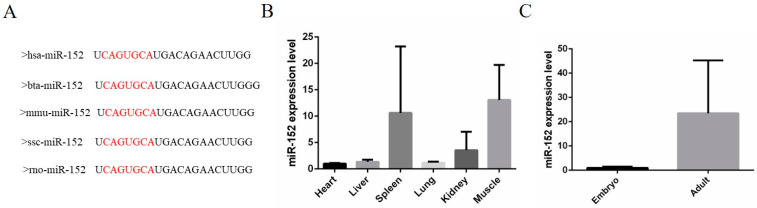
The expression characteristics of miR-152 in cattle. (**A**) The mature sequence of miR-152 in different species, hsa: human; bta: cattle; mmu: mouse; ssc: pig; rno: rat. The right letters shows miRNA seed sequence. (**B**) The expression feature of miR-152 in fetal bovine tissues, including heart, liver, spleen, lung, kidney, and muscles. (**C**) The expression level of miR-152 in fetal and adult bovine muscle tissues. *N* = 3.

MiR-152 belongs to the miR-148/152 family [18] and is often involved in a variety of cellular processes, including cell growth [19], apoptosis, and invasion [20]. In this study, the purpose of our experiment was to investigate the role of miR-152 in bovine muscle cell proliferation. Firstly, we performed a sequence conservative analysis of miR-152 in different species. There was no difference in the mature sequence of miR-152 between humans, cattle, mice, pigs, and rats (Figure 1A). Next, we investigated the expression features of miR-152 in different fetal calf tissues. The data showed that miR-152 is highly expressed in muscle tissues (Figure 1B). In addition, the expression level of miR-152 in fetal skeletal muscles was lower than in adult skeletal muscles (Figure 1C). Based on the above studies, we speculated that miR-152 might play a certain regulatory role in the development of bovine skeletal muscles.

### 3.2. miR-152 Regulates Bovine Myoblast Proliferation

**Figure 2:**
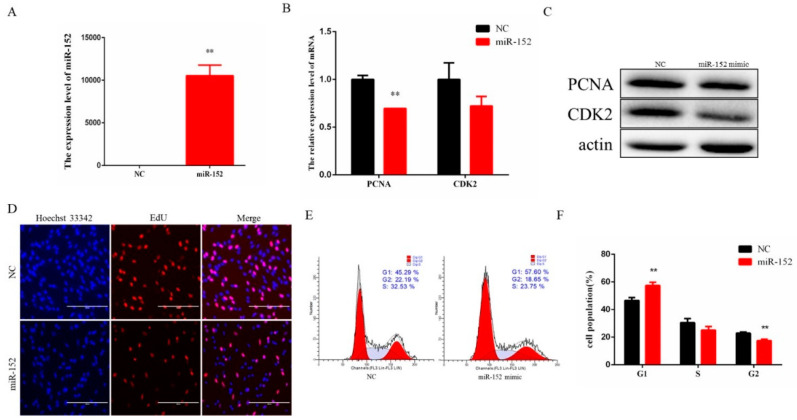
Overexpression of miR-152 inhibits bovine myoblast proliferation. (**A**) The expression level of miR-152 was detected by RT-qPCR after transfection of the miR-152 mimic. (**B**) The expression levels of PCNA and CDK2 were detected after transfection of the miR-152 mimic. (**C**) The protein expression levels of PCNA and CDK2 were detected by means of Western blotting after transfection of the miR-152 mimic (original western blot figures in Figure S1). (**D**) EdU-positive cells were detected after transfection of the miR-152 mimic. (**E**) Cell cycle was measured by means of flow cytometry after transfection of the miR-152 mimic. (**F**) Statistical results of the flow cytometry assay. The data are represented as the means ± SEM (*n* = 3); ** *p* < 0.01.

Muscle cell growth is the basis of meat yield, so it is of great significance to study the regulation of muscle cell proliferation. To investigate the biological role of miR-152 in bovine muscle cell proliferation, an exogenous miR-152 mimic was transfected into the cells to enhance its expression level. The expression level of miR-152 was significantly increased compared to the control group (Figure 2A). Meanwhile, we detected the mRNA and protein levels of PCNA and CDK2, which are key genes for cell proliferation. Compared to the control group, overexpression of miR-152 reduced the expression level of these genes (Figure 2B,C). In addition, we also examined the number of EdU-staining cells, and the data showed that the number of EdU-positive cells in the same field was significantly reduced compared with the control group (Figure 2D). Furthermore, cell cycle analysis by flow cytometry showed that the number of cells in the G_1_ phase was increased compared with the control group (Figure 2E,F).

**Figure 3:**
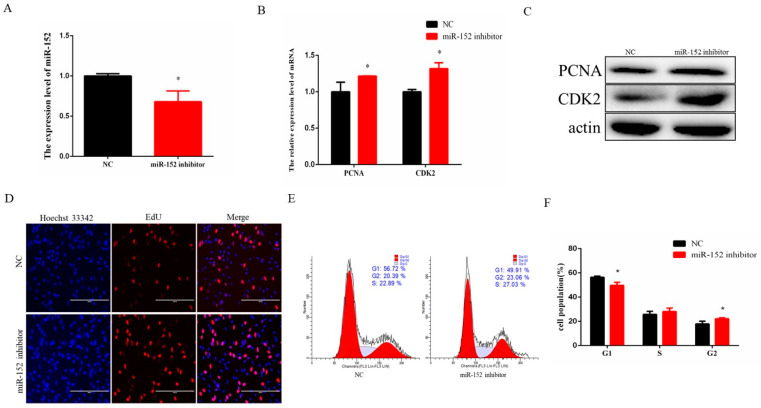
Inhibition of miR-152 promotes bovine myoblast proliferation. (**A**) The expression level of miR-152 was detected by RT-qPCR after transfection of the miR-152 inhibitor. (**B**) The expression levels of PCNA and CDK2 were detected after transfection of the miR-152 inhibitor. (**C**) The protein expression levels of PCNA and CDK2 were detected by means of Western blotting after transfection of the miR-152 inhibitor (original western blot figures in Figure S1). (**D**) EdU-positive cells were detected after transfection of the miR-152 inhibitor. (**E**) Cell cycle was measured by means of flow cytometry after transfection of the miR-152 inhibitor. (**F**) Statistical results of the flow cytometry assay. The data are represented as the means ± SEM (*n* = 3); * *p* < 0.05.

In order to verify the regulatory role of endogenous miR-152, we conducted a functional loss experiment with the miR-152 inhibitor. A synthetic miR-152 inhibitor was transfected into the cells to reduce its expression level significantly (Figure 3A). After the loss of miR-152, the mRNA expression level and the protein level of PCNA and CDK2 were increased (Figure 3B,C). The number of EdU-positive cells in the same field was significantly increased compared to the control group (Figure 3D). Cell cycle analysis by means of flow cytometry showed that the number of the G_1_ phase cells declined (Figure 3E,F). Taken together, these results suggested that endogenous miR-152 could inhibit bovine muscle cell proliferation.

### 3.3. KLF6 Is a Target Gene of miR-152

**Figure 4:**
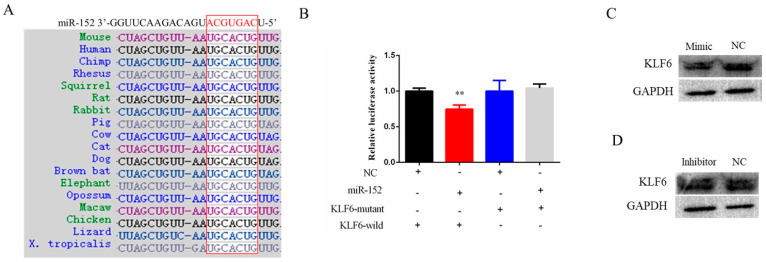
KLF6 is the target gene of miR-152. (**A**) The sequence of the miR-152 seed region targets the 3‘-UTR region of the KLF6 gene and is highly conserved in different species. (**B**) The relative luciferase activity assay was performed after co-transfection of the miR-152 mimic and the KLF6-3′-UTR wild-type or mutant-type dual-luciferase plasmid. (**C**) KLF6 protein expression level was detected by means of Western blotting after transfection of the miR-152 mimic (original western blot figures in Figure S2). (**D**) KLF6 protein expression level was detected by means of Western blotting after transfection of the miR-152 inhibitor (original western blot figures in Figure S3). The data are presented as the means ± SEM; ** *p* < 0.01.

To further explore the target gene of miR-152 in bovine muscle cells proliferation, online software Targetscan was used to target gene prediction, and the final results showed that KLF6 might be a potential target gene of miR-152 (Figure 4A). The prediction showed that the seed region sequence of miR-152 targets the 3’-UTR region of KLF6 and is highly conserved in different species. To further identify the targeting relationship between miR-152 and KLF6, firstly, we constructed a dual-luciferase reporter vector containing the 3’-UTR sequence (wild-type or mutant-type) of KLF6, respectively. Then, the dual-luciferase reporter vector (wild-type or mutant-type) and the miR-152 mimic were co-transfected into 293T cells, respectively. The results showed that the luciferase activity of the wild-type plasmid in the miR-152 group was decreased, but that of the mutant plasmid was increased (Figure 4B). Next, we detected the protein levels of KLF6 in bovine muscle cells after the gain or loss of miR-152. Compared with the control group, the protein level of KLF6 was decreased after the gain of miR-152 and increased after the loss of miR-152 (Figure 4C,D). Together, these results suggested that KLF6 is a target gene of miR-152.

## 4. Discussion

As previously mentioned, numerous studies on skeletal muscle cell proliferation have been reported, certainly including the regulation of miRNAs as shown in the review. In addition to these miRNAs, there are still many miRNAs the functions and mechanisms whereof regarding regulation of the skeletal muscle development remain to be elucidated. In this study, we investigated the expression characteristics of miR-152 in bovine tissues, and the results showed that miR-152 was highly expressed in muscle tissues. Further functional and mechanism studies showed that miR-152 inhibited bovine muscle cell proliferation by targeting KLF6.

This miRNA, miR-152, has the same “seed region” as miR-148a, a tumor-associated miRNA which was reported to be involved in tumorigenesis [18]. In endometrial cancer, miR-152 inhibited tumor cell growth via targeting novel candidate targets E2F transcription factor 3 (E2F3), tyrosine kinase receptor (MET), and rapamycin-insensitive companion of mTOR (Rictor) [21]. In epithelial ovarian cancer, miR-152 functioned through suppressing DNA methyltransferase 1 (DNMT1) to inhibit ovarian cancer cell proliferation and promote apoptosis [22]. In glioblastoma (GBM), overexpression of miR-152 reduced glioblastoma stem cell proliferation, migration, and invasion, as well as induced apoptosis via targeting Krüppel-like factor 4 (KLF4) [23]. In prostate cancer, miR-152 reduced the prostate cancer cells’ migratory and invasive capabilities through directly targeting TGFα [20]. In addition, the expression level of miR-152 in serum can act as a novel biomarker in non-small-cell lung cancer screening [24]. Most of the previous studies focused on miR-152 as a tumor-suppressive microRNA, and detailed literature reports were reviewed [18]. As mentioned above, miR-152 mainly participates in tumorigenesis through targeting different target genes.

During the mammary gland development, miR-152 enhances cow mammary epithelial cell proliferation, inhibits apoptosis, and increases triglyceride production through downregulating the mRNA and protein levels of acetyl-CoA acyltransferase 2 (ACAA2) and hydroxysteroid 17-beta dehydrogenase 12 (HSD17B12) [25]. RNA sequencing results showed that miR-152 is highly expressed in porcine skeletal muscles [26]. A previous study reported that miR-152 inhibits C2C12 myoblast proliferation through targeting E2F3 [27]. However, there are few reports about the function of miR-152 in livestock animal skeletal muscle development. In this study, we found that miR-152 is highly expressed in bovine skeletal muscle tissues. Functionally, through RT-qPCR, Western blotting, EdU assay, and cell cycle assay, the results show that miR-152 inhibits bovine muscle cell proliferation. Mechanically, through the dual-luciferase reporter assay and the Western blot analysis, the data indicated that KLF6 is a direct target gene of miR-152. KLF6 is a zinc finger transcription factor involved in a variety of biological processes [28,29], including skeletal muscle development. In a previous study, knockdown of KLF6 was reported to inhibit C2C12 cell proliferation [30]. We also demonstrated that KLF6 could promote bovine myoblast proliferation [31]. The function of KLF6 for myoblast proliferation was opposite to that of miR-152, which is consistent with this research.

## 5. Conclusions

In conclusion, our study found that miR-152 functions in bovine myoblasts to inhibit proliferation. Our prediction and experimental verification revealed that KLF6 is a direct target gene of miR-152. Therefore, miR-152 and its target gene KLF6 had certain effects on the development of bovine skeletal muscles.

## Data Availability

Data is contained within the article or Supplementary Material.

## Supplementary Materials

The following are available online at https://www.mdpi.com/article/10.3390/ani11103001/s1, Figure S1: Original western blot figures for Figure 2C and Figure 3C, Figure S2: Original western blot figures for Figure 4C, Figure S3: Original western blot figures for Figure 4D, Table S1: Primers sequence for PCR and RT-qPCR, Table S2: The primary antibody and the secondary antibody.
