# Supplementary material for: miR-152 Regulates Bovine Myoblast Proliferation by Targeting KLF6
Source: Animals (Basel). 2021 Oct 19;11(10):3001. doi: 10.3390/ani11103001 (PMC8532817; doi:10.3390/ani11103001)
Supplement: Supplementary file 1 [file animals-11-03001-s001.zip › animals-1413126-supplementary.pdf]

Table S1. Primers sequence for PCR and RT-qPCR

Name	Sense Primers	Antisense Primers
GAPDH	TGAGGACCAGGTTGTCTCCTGCG	CACCACCCTGTTGCTGTAGCCA
ACTB	CTGGAACGGTGAAGGTGACA	AATGGACTTCCTGTAACAATGCA
PCNA	TCCAGAACAAGAGTATAGC	TACAACAGCATCTCCAAT
CDK2	TTTGCTGAGATGGTGACCCG	TAACTCCTGGCCAAACCACC
miR-152-RT	GTCGTATCCAGTGCAGGGTCCGAGGTATTCGCACTGGATACGACCCCAAGT	
miR-152	GGCGGTCAGTGCATGACA	CCAGTGCAGGGTCCGAGGTA
psiCHECK2-KLF6	CCG**<u>CTCGAG</u>**CTGCACATGAAGAGACACCTCTG	ATAAGAAT**<u>GCGGCCGC</u>**TTCTCAGTGCACATGCTCACGGC
psiCHECK2-KLF6-Mutant	CCG**<u>CTCGAG</u>**CTGCACATGAAGAGACACCTCTG	ATAAGAAT**<u>GCGGCCGC</u>**TTCTCAGACCACATGCTCACGGC

Table S2. The primary antibody and secondary antibody.

Antibody name	Purpose	Source
Actin	primary antibody	Abbkine
CDK2	primary antibody	Sangon Biotech
PCNA	primary antibody	Sangon Biotech
KLF6	primary antibody	bioss
GAPDH	primary antibody	Sangon Biotech
Goat Anti-Rabbit	secondary antibody	BOSTER

a

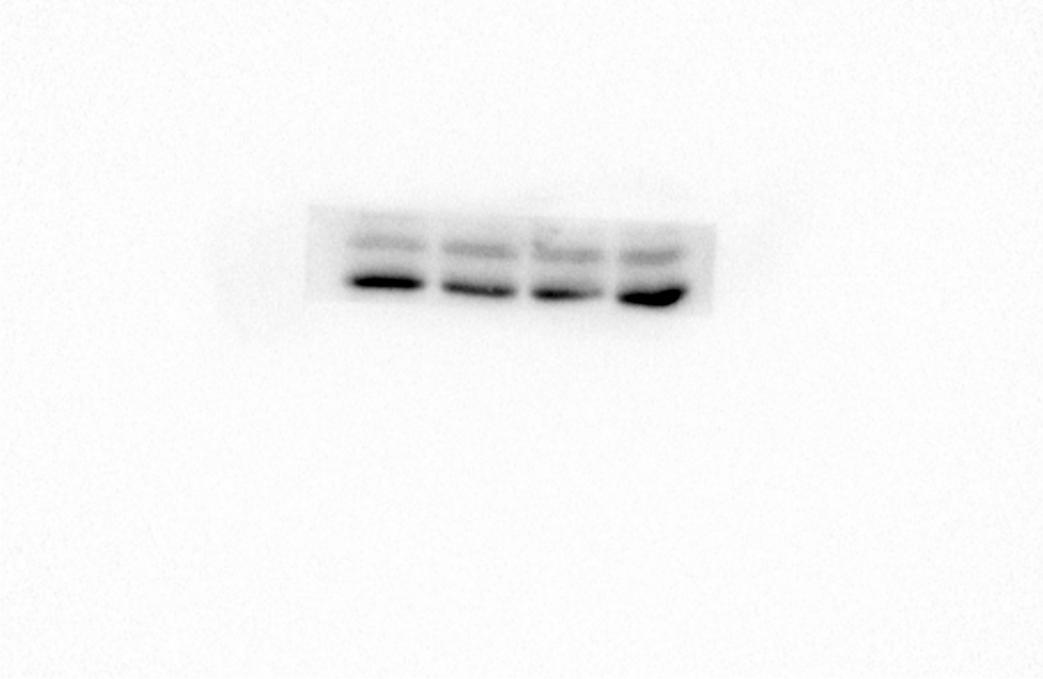

b

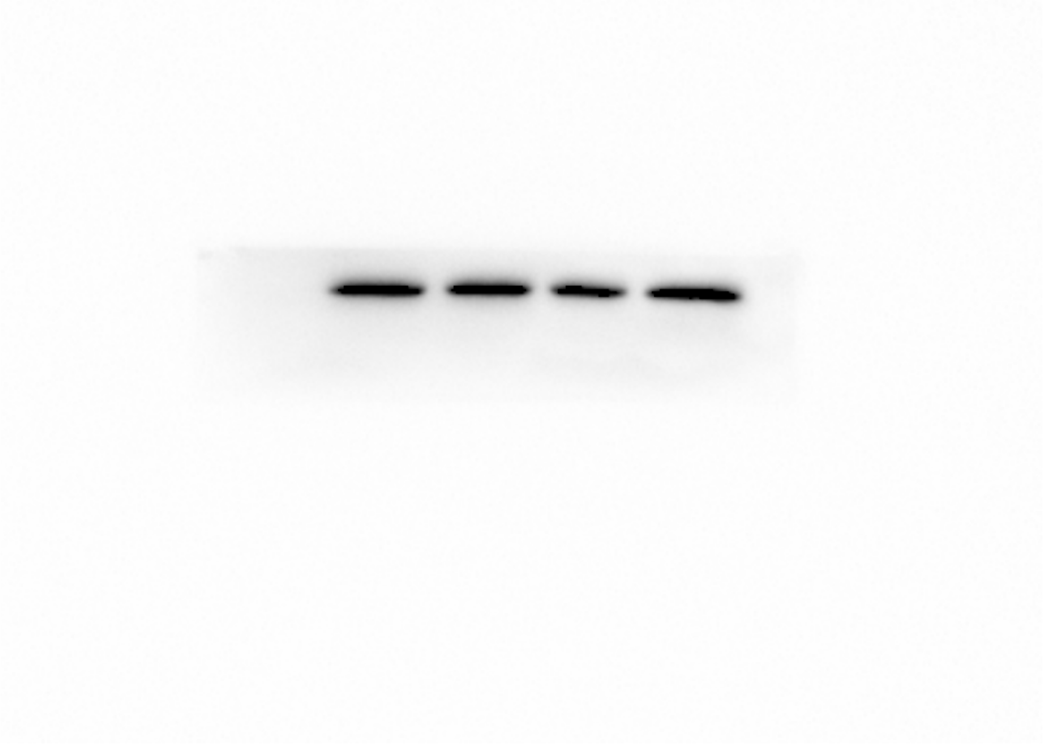

c

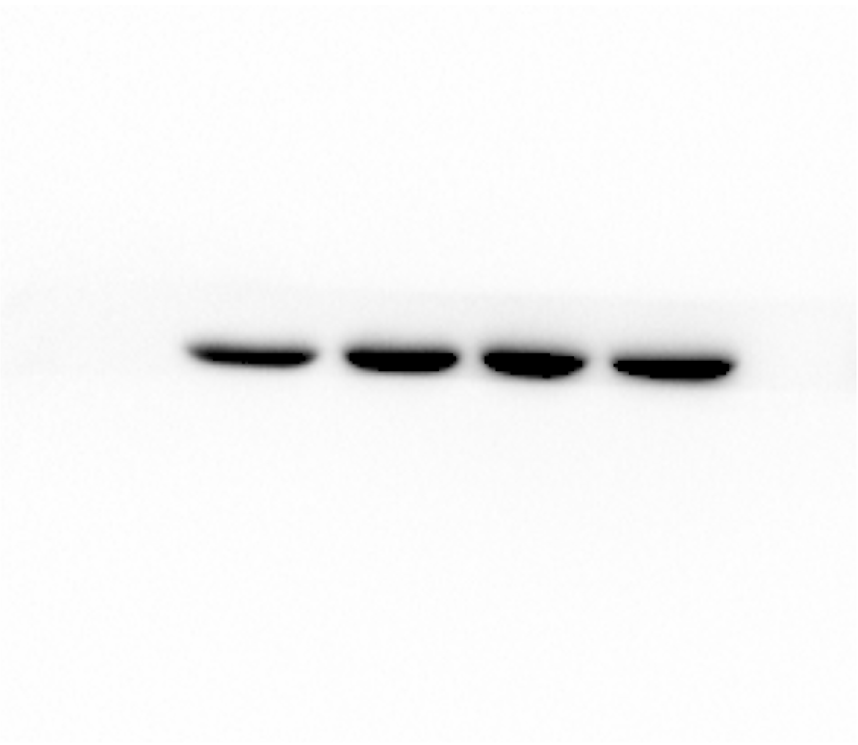

Figure S1. Original western blot figures for Figure2C & 3C. (**a**) CDK2, Left to right NC, miR-152 mimic, NC, miR-152 inhibitor. (**b**) PCNA, Left to right NC, miR-152 mimic, NC, miR-152 inhibitor. (**c**) Actin.

a 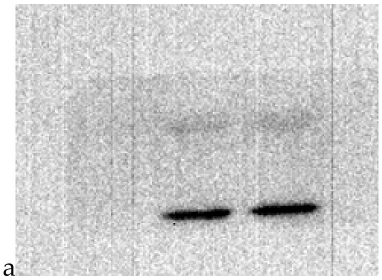 b 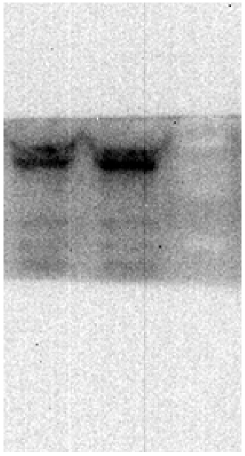

Figure S2. Original western blot figures for Figure4C. Right to left: NC miR-152 mimic (**a**) GAPDH; (**b**) KLF6.

a 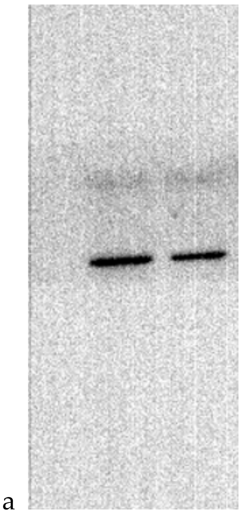 b 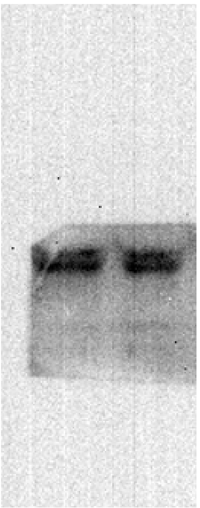

Figure S3. Original western blot figures for Figure4D. Right to left: NC miR-152 inhibitor. (**a**) GAPDH; (**b**) KLF6.
